# Maleic Acid-Grafted Poly(vinylpyrrolidone) Hydrogels Synthesized by Gamma Radiation for pH-Responsive Drug Delivery

**DOI:** 10.3390/gels12080739

**Published:** 2026-08-18

**Authors:** Miguel S. Pérez-Garibay, Emilio Bucio

**Affiliations:** Departamento de Química de Radiaciones y Radioquímica, Instituto de Ciencias Nucleares, Universidad Autónoma de México, Circuito Exterior, Ciudad Universitaria, Coyoacán, Mexico City 04510, Mexico

**Keywords:** PVP, maleic acid, hydrogels, γ-ray, naringin, benzalkonium chloride, antimicrobial silver, pH response

## Abstract

Poly(vinylpyrrolidone) hydrogels net(PVP) were synthesized by gamma radiation at a dose rate of 11.8 kGy h^−1^ and subsequently functionalized with maleic acid (MA) via radiation-induced grafting using the direct method to obtain pH-responsive hydrogels for the loading and controlled release of bioactive compounds. Under the selected conditions, MA grafting reached approximately 18%. The obtained hydrogels were characterized by Fourier transform infrared spectroscopy (FTIR-ATR), differential scanning calorimetry (DSC), thermogravimetric analysis (TGA), and swelling studies. The results confirmed the successful incorporation of MA into the hydrogel network and demonstrated that grafting imparted pH-responsive behavior due to the ionization of carboxylic acid groups, yielding two critical pH values at 4.4 and 6.0. Loading and release studies using naringin and benzalkonium chloride showed that the grafted hydrogels exhibited enhanced loading capacity and sustained release profiles governed by the hydrogel network and by interactions between the loaded compounds and the carboxylic groups from MA. Furthermore, hydrogels loaded with antimicrobial agents effectively inhibited the growth of *Escherichia coli* and *Staphylococcus aureus*. These findings demonstrate that gamma radiation-induced MA grafting is an effective strategy for developing pH-responsive PVP hydrogels with potential applications as multifunctional wound dressing materials.

## 1. Introduction

Hydrogels are highly relevant materials due to their unique properties, such as softness, flexibility, and biocompatibility [1]. Structurally, hydrogels are three-dimensional molecular networks linked by covalent or non-covalent bonds [2]. Due to the presence of polar groups in their structure, such as SO_3_H, OH, NH_2_, COOH, and CONH_2_, they can absorb considerable amounts of water or biological fluids, maintaining network integrity while remaining in a swollen state [3]. Therefore, they are widely studied in various fields, including agriculture, biomaterials, the food industry, drug delivery, tissue engineering, and regenerative medicine [4,5]. Hydrogels are typically classified into two categories: physically cross-linked and chemically cross-linked, which exhibit different properties depending on the synthesis method [6]. Physically crosslinked hydrogels can be reversibly formed or disrupted due to non-covalent interactions. These interactions can be of different nature such as hydrophobic interactions, hydrogen bonds, electrostatic interactions, or crystallized segments [6,7].

Chemically crosslinked hydrogels, on the other hand, contain permanent covalent crosslinks [7]. These covalent networks can be generated through different approaches, including radical-mediated crosslinking, high-energy irradiation, and appropriate chemical crosslinking reactions, among other methods [6,8]. To improve properties such as electrical conductivity, mechanical performance, and responsiveness to specific stimuli, various additives or polymers can be incorporated into hydrogels [9]. In this context, grafting functional monomers onto hydrogels introduces polymer chains with distinct physicochemical characteristics, including hydrophilic and hydrophobic segments [10]. This modification allows the development of hydrogels responsive to a variety of external stimuli, thereby broadening their potential applications in diverse fields [11,12]. The grafted polymer chains are generally flexible and can undergo rapid conformational changes in response to environmental changes (h35).

This behavior has been exploited to increase both the swelling capacity and swelling rate of hydrogels, as well as to accelerate the contraction–swelling response of stimulus-responsive hydrogels under changing external conditions [10,13].

On the other hand, the gamma radiation technique stands out as one of the predominant methods for crosslinking and grafting in various polymer blends and composite systems [14]. In addition, gamma radiation enables polymer synthesis and grafting without the need for chemical initiators or crosslinking agents, allowing the simultaneous production and sterilization of highly pure materials in a single step, which is particularly advantageous for biomedical applications, while the radiation dose can be adjusted to tailor key properties such as gel content and swelling behavior [15,16].

Poly(N-vinylpyrrolidone) (PVP) is a synthetic polymer widely investigated for the preparation of hydrogels and other platforms intended for biomedical applications [17].

Its biocompatibility, low toxicity, chemical stability, hydrophilicity, water solubility, and resistance to a broad range of biofouling compounds make it particularly attractive for biomedical and pharmaceutical uses [18,19]. Furthermore, PVP-based hydrogels exhibit favorable properties such as transparency and good biocompatibility [20], while the polymer’s broad solubility and ability to form complexes with a wide variety of low-molecular-weight compounds further expand its potential as a matrix for incorporating and delivering bioactive agents [21].

Conversely, maleic acid (MA) has recently attracted considerable interest in polymer synthesis [22]. It has been combined with other compounds to produce various materials, including copolymers [23,24,25,26], hydrogels [27,28,29,30], nanoparticles, and microparticles [31], resulting in biocompatible and pH-responsive materials [22,27,28,29,30]. Owing to these properties, maleic acid has been investigated for biomedical applications, particularly in wound dressings [32,33].

On the other hand, naringin (NA) is a flavanone glycoside found in citrus fruits and is primarily responsible for the bitterness of their juice [34]. NA loading and release were investigated due to its reported antioxidant, antitumor, anti-inflammatory, antimicrobial, and biocompatible properties [35,36,37,38]. Furthermore, NA induces platelet aggregation [39,40] and promotes angiogenesis, thereby accelerating wound healing [41,42,43,44].

Benzalkonium chloride (Bz-Cl) and antimicrobial silver were studied as antimicrobial agents. Bz-Cl belongs to a class of quaternary ammonium compounds known as alkyldimethylbenzylammonium chlorides. It is generally marketed as a mixture of homologs with alkyl chain lengths ranging from C_8_ to C_18_ [45]. The biocidal activity of benzalkonium chloride depends on the alkyl chain length, with the C_12_ and C_14_ homologs exhibiting the highest antimicrobial efficacy [45]. Owing to its antimicrobial properties, Bz-Cl is widely used in medical applications [46]. It can inhibit the proliferation of clinically relevant microorganisms, including *Pseudomonas aeruginosa*, *methicillin-resistant Staphylococcus aureus*, and vancomycin-resistant *Enterococcus* species [47]. Specifically, Bz-Cl has been used to treat wounds and burns, exhibiting 92% effectiveness against *E. coli* and 96% against *S. aureus* [48]. In in vivo studies, Bz-Cl loaded hydrocolloids were shown to accelerate wound healing in mice [49].

Meanwhile, antimicrobial silver exhibits inhibitory and bactericidal activity against a broad spectrum of microorganisms, including bacteria, fungi, and viruses [50]. Owing to these properties, it has been extensively studied for decades as an antimicrobial agent for a wide range of applications, including the dental, medical, pharmaceutical, textile, and fiber sectors [50,51]. Additionally, silver nanoparticles represent a promising alternative for controlling multidrug-resistant bacteria [51,52]. Furthermore, they have been reported to retain their antimicrobial activity under different temperature and pH conditions, thereby broadening their potential use in diverse environments and applications [53].

Although PVP-based hydrogels, materials containing maleic acid, and antimicrobial silver have been studied separately for biomedical applications, integrating them into a gamma-radiation-functionalized PVP hydrogel is of particular interest. This approach incorporates two ionizable carboxyl groups into the PVP network, yielding materials with two critical pH values within the skin’s pH range. Furthermore, evaluating compounds with diverse physicochemical properties—such as NA, Bz-Cl, and antimicrobial silver—provides insight into how the chemical environment created by maleic acid (MA) grafting influences loading and release behavior. In this context, and to expand the range of potential strategies for burn treatment, PVP hydrogels (net(PVP)) were synthesized and subsequently grafted with maleic acid net(PVP)-g-MA. These hydrogels were then loaded with NA and antimicrobial agents for controlled release, aiming to develop pH-responsive materials that prevent infections and promote faster tissue regeneration.

## 2. Results and Discussion

### 2.1. Obtention of net(PVP) Hydrogels

Table 1 presents the gel content values for hydrogels synthesized from PVP or NVP in water or methanol at different irradiation doses. The results for the PVP-based hydrogels (360 kDa) indicate that both polymer concentration and irradiation dose significantly influenced the gel content.

For formulations containing 10% PVP in water, the gel content increased from 54 to 73% as the irradiation dose increased from 10 to 40 kGy. When the PVP concentration in water was increased to 15%, gel contents of 78 and 81% were obtained at doses of 25 and 50 kGy, respectively. These results may be attributed to the greater proximity between macromolecular chains at higher polymer concentrations, which increases the probability of radical recombination and consequently promotes the formation of an insoluble crosslinked fraction [54].

**Figure 1:**
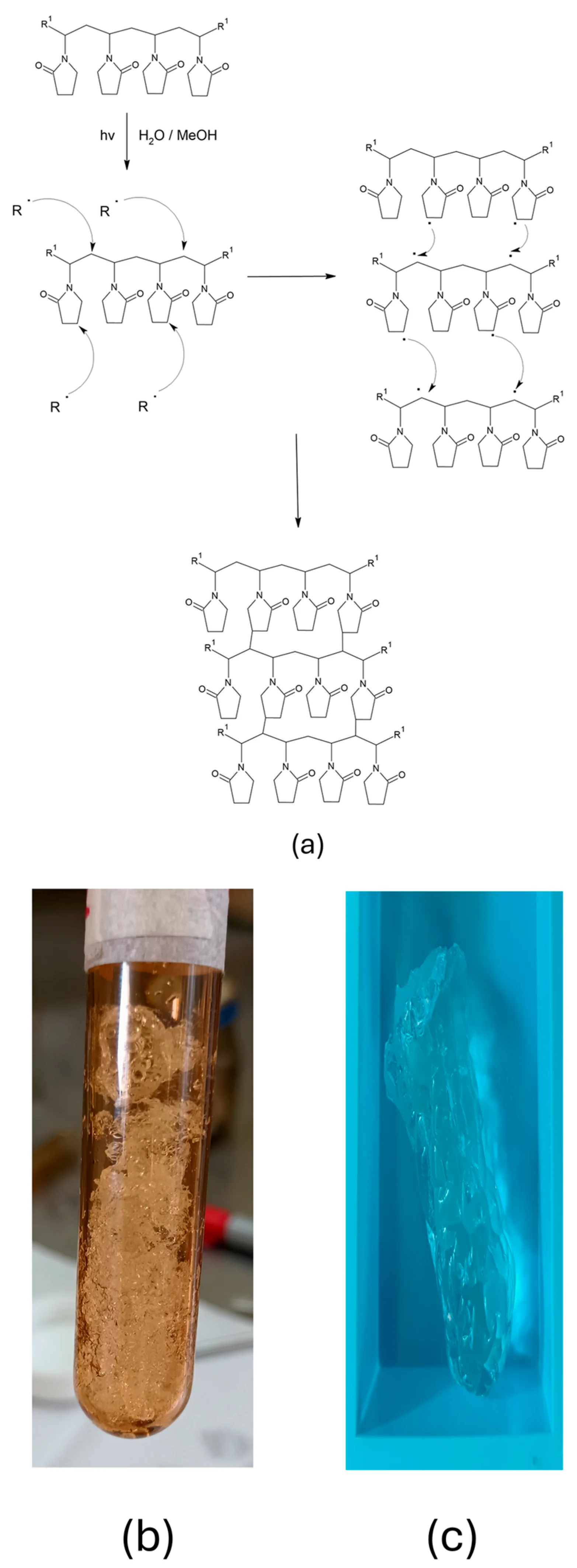
(**a**) Proposed mechanism for the synthesis of hydrogel net(PVP). (**b**) Hydrogel net(PVP) obtained from PVP 360 kDa at 40 kGy. (**c**) Hydrogel net(PVP) obtained from NVP monomer at 40 kGy.

In contrast, samples prepared with 15% PVP in methanol exhibited lower gel content values of approximately 62–63%, regardless of the applied dose. This behavior may be associated with several factors, including methanol’s ability to act as a free-radical scavenger [55,56] and its lower polarity compared with water, which may affect the solvation of PVP and limit the mobility and expansion of its polymer chains during network formation [57]. Furthermore, gamma irradiation of water generates several reactive radical species [58,59,60] that promote hydrogen abstraction from the carbon backbone of PVP (Figure 1a). The resulting macroradicals can subsequently recombine, leading to the formation of a three-dimensional polymer network and increasing the insoluble gel fraction (PVP).

However, the PVP-based hydrogels exhibited poor handling characteristics, as they appeared fragile, brittle, and insufficiently flexible during their removal from the ampoules (Figure 1b). These observations were based exclusively on qualitative assessment by touch and handling rather than on quantitative mechanical measurements. This behavior is hypothesized to result from the strong solvation of PVP chains and other polymers containing hydrophilic groups in aqueous media, which promotes relatively extended chain conformations. Consequently, the radiation-induced network may exhibit a lower crosslinking density and greater swelling capacity [61,62], potentially resulting in limited elasticity and mechanical strength. Nevertheless, this interpretation must be corroborated in future studies through quantitative mechanical tests, such as tensile, compression, or rheological measurements.

Based on these findings, systems formulated with 50% (*w*/*w*) NVP monomer in methanol were subsequently evaluated because they exhibited the highest gel content values. At 20 kGy, hydrogels with a low gel content were obtained, indicating that this dose was insufficient to promote extensive monomer polymerization and subsequent network formation. Increasing the dose to 40 kGy produced a maximum gel content of 95%. However, this value decreased to 83% at 60 kGy, suggesting that polymer degradation may have occurred at the higher dose, as previously reported for PVP hydrogels [63]. In particular, the hydrogels obtained at 40 and 60 kGy appeared more compact and malleable during handling, which may be associated with the formation of a more homogeneous and highly crosslinked polymer network (Figure 1c). However, these characteristics were assessed only qualitatively by touch and handling. Therefore, mechanical testing will be required in future studies to confirm these preliminary observations. Based primarily on their high gel content and favorable handling characteristics, the hydrogels synthesized at 40 kGy were selected for subsequent treatments.

We hypothesized that the differences in gel content and qualitative handling characteristics between hydrogels prepared from PVP (360 kDa) and those synthesized from the NVP monomer may be explained by their distinct network-formation mechanisms. When preformed PVP is used, the mobility of the high-molecular-weight polymer chains is limited, and irradiation can simultaneously induce crosslinking and chain-scission reactions, potentially resulting in partial polymer degradation and a lower insoluble fraction [17]. In contrast, hydrogels synthesized from NVP undergo simultaneous radiation-induced polymerization and crosslinking, favoring the formation of a more homogeneous and compact three-dimensional network and consequently producing a higher gel content and apparently better handling characteristics [64]. Nevertheless, future quantitative mechanical testing is necessary to determine whether these qualitative differences correspond to actual improvements in mechanical performance.

### 2.2. Obtention of net(PVP)-g-MA Hydrogels

**Figure 2:**
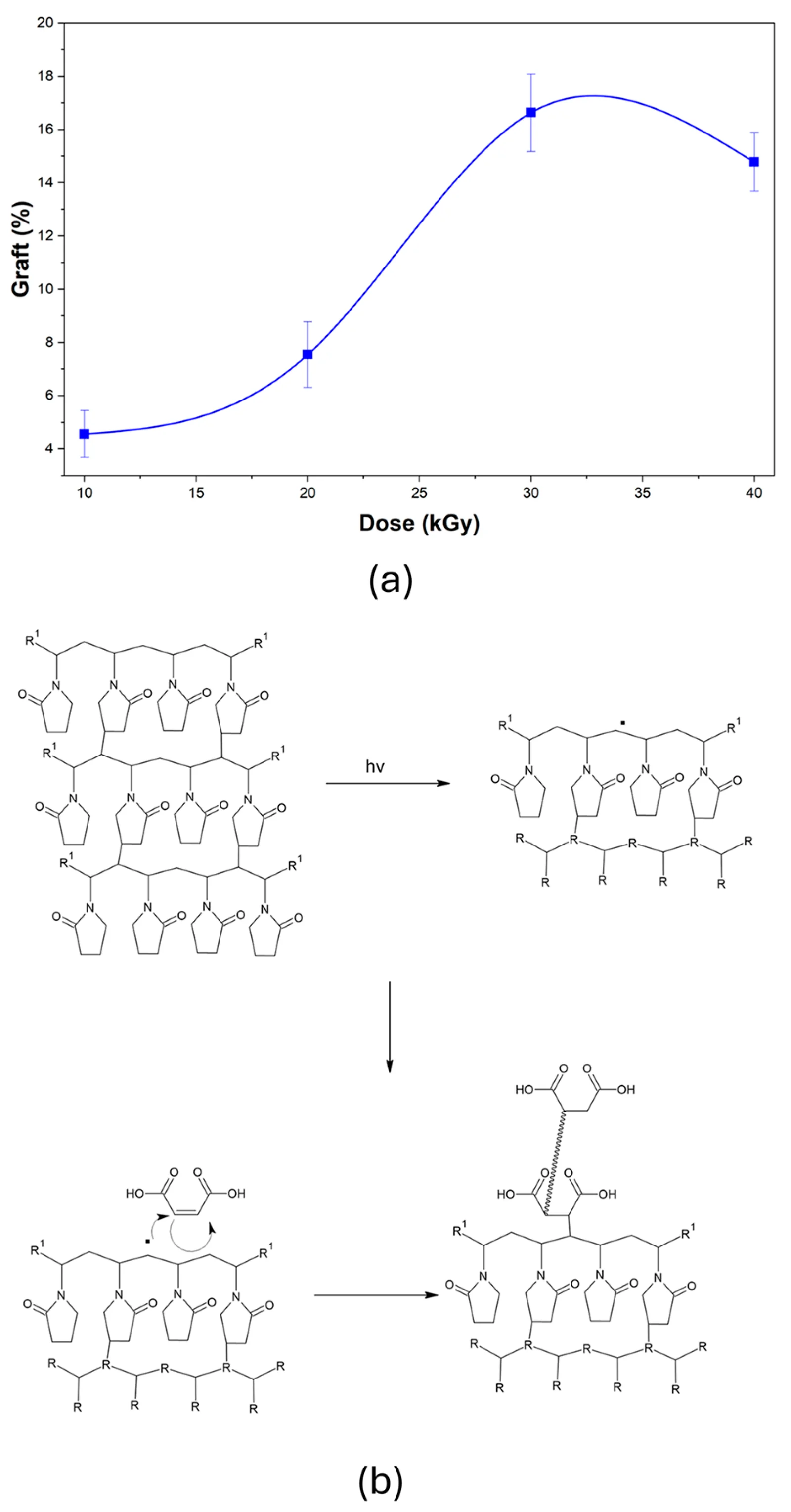
(**a**) Grafting of MA onto net(PVP) hydrogels varying dose. (**b**) Proposed mechanism for grafting of MA onto net(PVP).

Figure 2a shows the grafting percentages of MA onto net(PVP) hydrogels. The grafting percentage increased with irradiation dose, from approximately 4.5% at 10 kGy to 16.6% at 30 kGy, then decreased to 14.7% at 40 kGy. At low irradiation doses, the grafting percentage was relatively low because fewer free radicals capable of reacting with MA were generated along the PVP chains. Increasing the dose to 20 and 30 kGy promoted the formation of additional active sites within the polymer matrix, thereby enhancing MA grafting [65]. However, at 40 kGy, the grafting percentage decreased. This behavior may be attributed to competition between grafting and side reactions, including polymer degradation and radical recombination [66]. Based on the maximum grafting percentage obtained, the hydrogels prepared at 40 kGy were selected for subsequent characterization. The proposed mechanism for MA grafting onto the net(PVP) is presented in Figure 2b. It should be noted that only MA grafting onto a single carbon is shown for practical reasons; however, grafting can occur along the entire carbon chain, and MA cross-linking is also possible.

### 2.3. Obtention of Antimicrobial Silver

**Figure 3:**
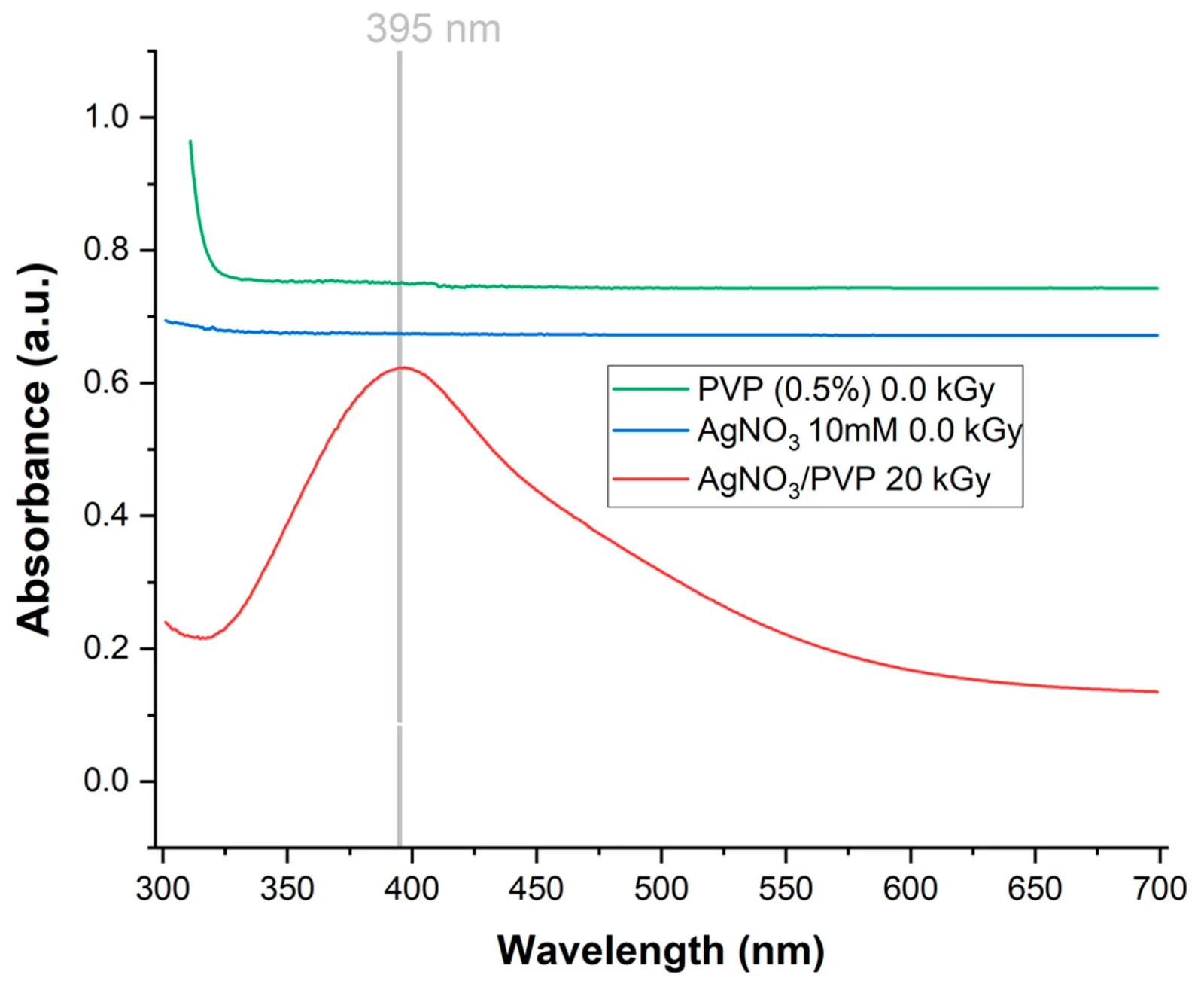
UV spectra of AgNPs/PVP obtained by γ-ray irradiation reduction of silver nitrate.

Separately from the hydrogel synthesis described above, antimicrobial silver was prepared by gamma irradiation of a PVP/AgNO_3_ solution at 20 kGy for its subsequent incorporation into the hydrogels as an antimicrobial agent. Before irradiation, the PVP/AgNO_3_ solutions were transparent and colorless. After irradiation, the samples turned brown, indicating the formation of silver nanoparticles (AgNPs) and silver oxide nanoparticles, consistent with the employed methodology [67,68]. UV–Vis spectroscopy revealed a maximum absorption band at 395 nm (Figure 3), close to the surface plasmon resonance reported in the literature at approximately 400 nm [69,70]. However, UV–Vis spectroscopy alone does not allow the particle size distribution or morphology to be conclusively determined. These parameters should be evaluated through complementary characterization in future characterizations such as transmission electron microscopy (TEM), atomic force microscopy (AFM), or dynamic light scattering (DLS). PVP was incorporated into the colloidal dispersion because it has been reported to promote AgNPs nucleation and to act as an effective stabilizing agent, preventing agglomeration and maintaining the nanoparticles dispersed in the medium [71].

### 2.4. Fourier Transform Infrared Spectroscopy with Attenuated Total Reflectance (FTIR-ATR)

**Figure 4:**
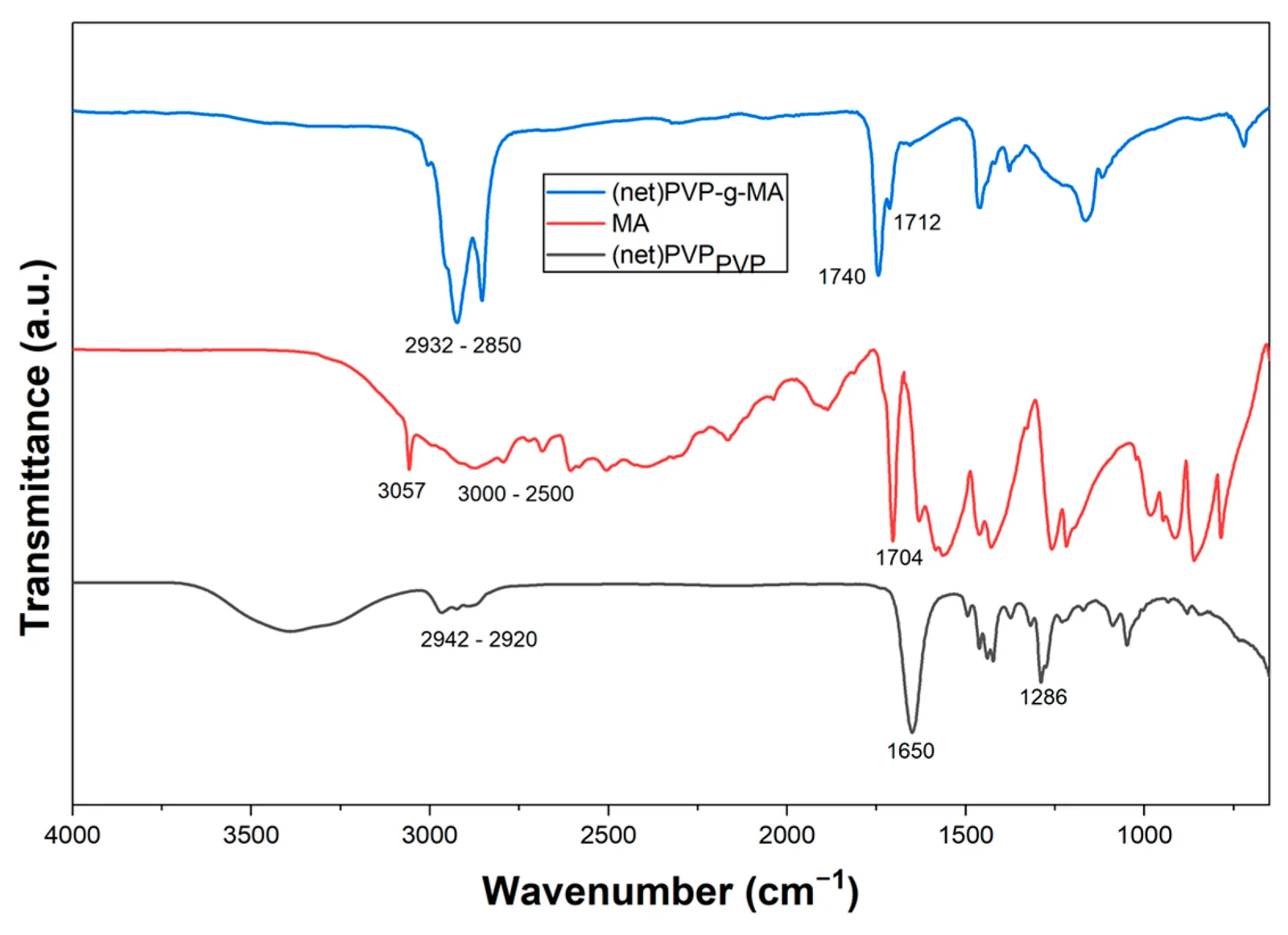
FTIR-ATR analysis of net(PVP), MA and net(PVP)-g-MA.

Figure 4 shows the FTIR spectra of the net(PVP) and net(PVP)-g-MA hydrogels, as well as that of the MA monomer. The net(PVP) hydrogel exhibits bands at approximately 2942–2920, 1650, and 1286 cm^−1^, attributed to C–H stretching of the polymer backbone, C=O stretching of the carbonyl group, and C–N stretching, respectively [18,21,72].

The MA monomer exhibits a band at approximately 3057 cm^−1^, attributed to the stretching of sp^2^ C–H bonds, as well as a broad band in the 3000–2500 cm^−1^ region associated with O–H stretching of the carboxylic acid groups. In addition, the intense band at approximately 1704 cm^−1^ is assigned to C=O stretching of these groups [28,32,73].

For the net(PVP)-g-MA hydrogel, corresponding to the hydrogel samples prepared at an irradiation dose of 40 kGy. In this case, a notable increase in the intensity of the bands at approximately 2932 and 2850 cm^−1^ is observed, corresponding to the asymmetric and symmetric stretching vibrations of aliphatic C–H bonds, respectively. This behavior may be associated with changes in the chemical environment and conformation of the PVP network after MA grafting. Furthermore, the bands at approximately 1740 and 1712 cm^−1^, assigned to carbonyl groups, support the incorporation of MA into the hydrogel network [74]. The absence of a well-defined band at approximately 1286 cm^−1^, attributed to the characteristic C–N stretching vibration of PVP, could result from changes in its intensity or overlap with other signals following modification of the polymer network. Previous studies have demonstrated that PVP-based systems containing maleic acid-derived compounds can establish strong intermolecular interactions through hydrogen bonding, resulting in shifts and changes in the characteristic FTIR bands of PVP [75].

### 2.5. Differential Scanning Calorimetry (DSC)

DSC is a widely used thermoanalytical technique for polymer characterization because it allows the thermal transitions experienced by a material to be evaluated as a function of temperature. This technique enables the identification of characteristic transitions, including the glass transition temperature (Tg), melting temperature (Tm), and crystallization temperature (Tc), among others [76].

**Figure 5:**
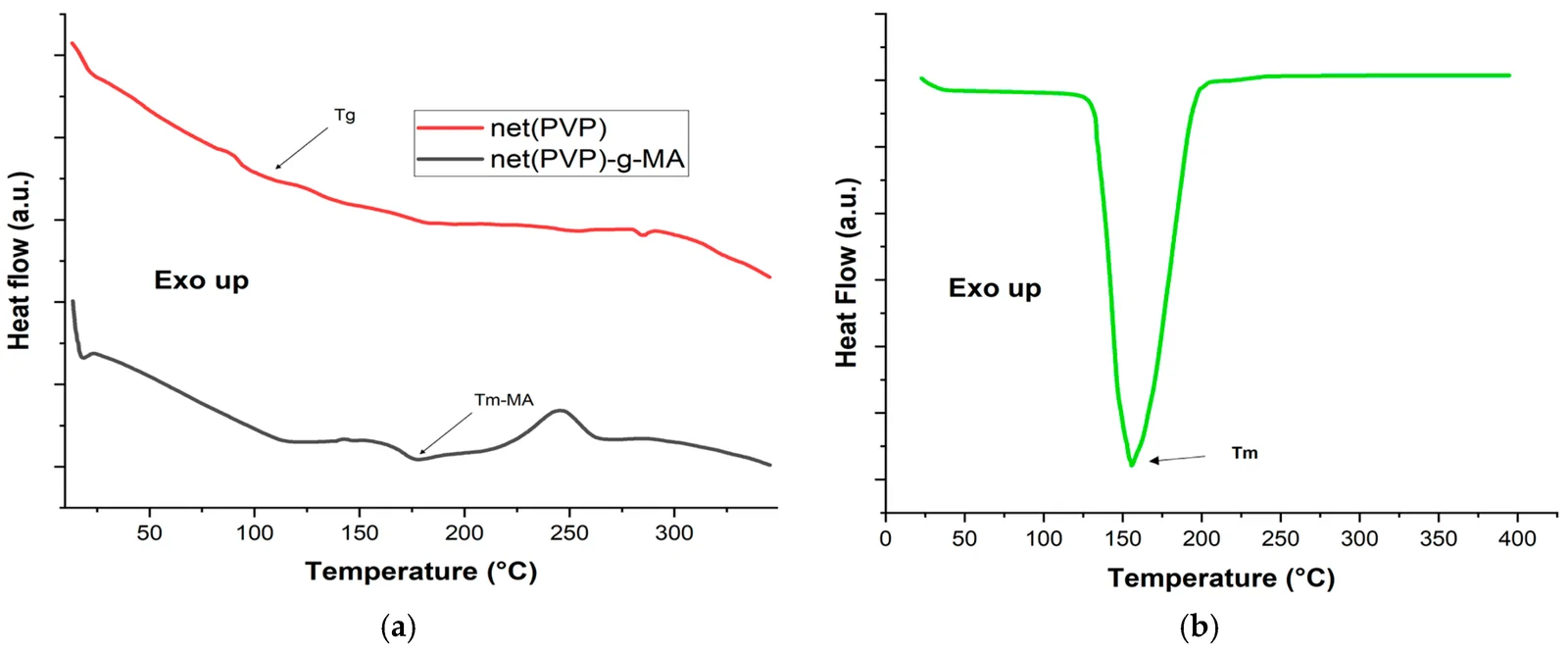
(**a**) DSC analysis of net(PVP) and net(PVP)-g-MA. (**b**) DSC of MA monomer.

For the net(PVP) hydrogel (Figure 5a), a Tg of approximately 105 °C was observed. Tg values ranging from 113 to 180 °C have been reported for PVP [77]. These variations have been attributed to differences in molecular weight and polymerization method [77]. In addition, the thermal event observed at approximately 280 °C may be associated with the onset of PVP degradation. The MA monomer (Figure 5b) exhibits a melting temperature (Tm) of approximately 155 °C, whereas values ranging from 137 to 197 °C have been reported in the literature [23,78]. Furthermore, the net(PVP)-g-MA (Figure 5a) hydrogel exhibits an endothermic peak at approximately 177 °C. This additional thermal event supports the incorporation of MA into the polymer network. Its shift relative to the melting peak of the pure monomer may be associated with changes in the chemical environment of MA after grafting, including intermolecular interactions between the carboxylic acid groups of MA and the carbonyl groups of PVP.

### 2.6. Thermogravimetric Analysis (TGA)

TGA is a widely used technique for polymer characterization because it allows changes in a material’s mass to be evaluated as a function of temperature or time. These changes may result from decomposition and oxidation reactions, as well as physical processes such as sublimation, vaporization, and desorption [79].

**Figure 6:**
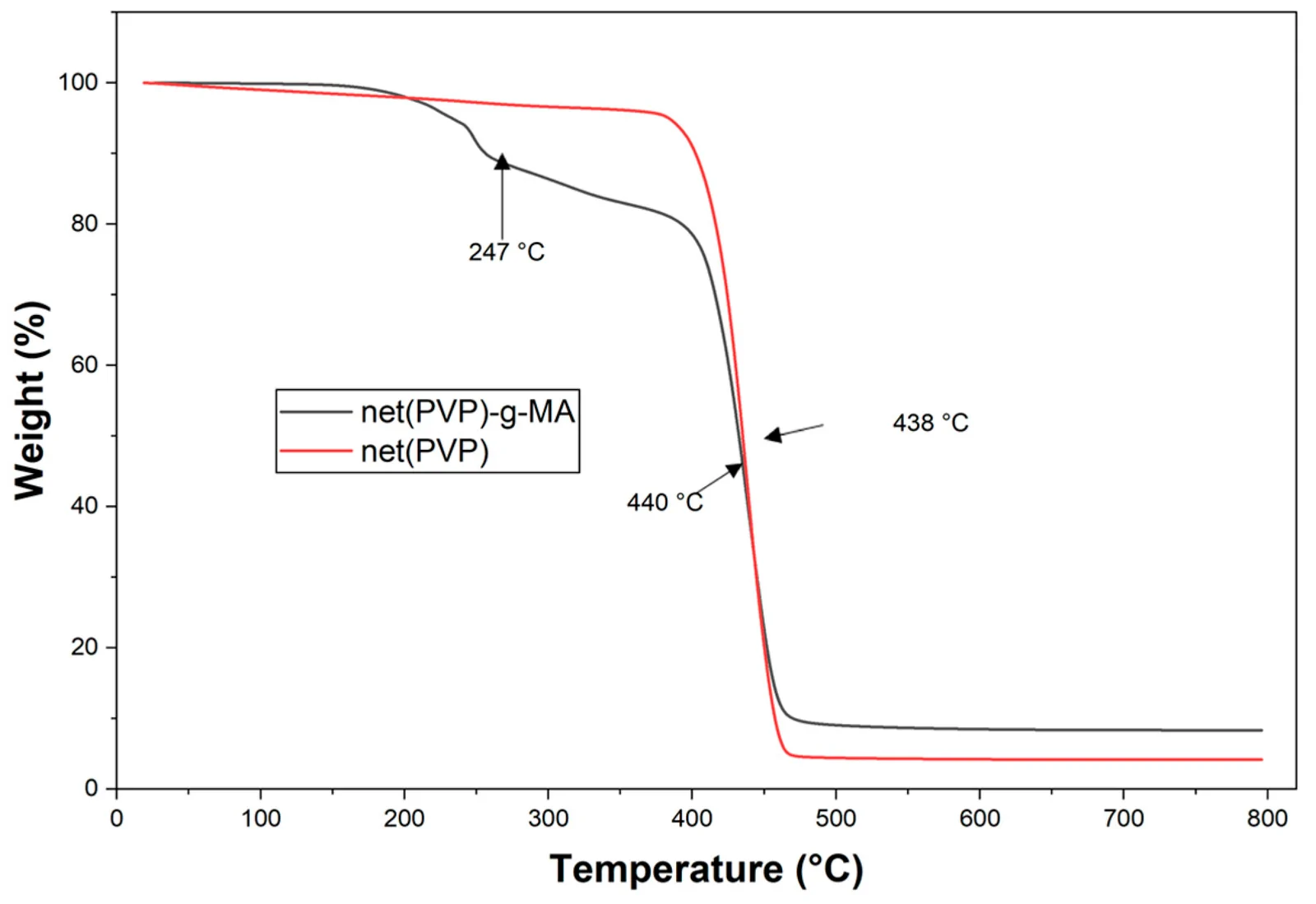
TGA of net(PVP) and net(PVP)-g-MA.

The thermogram of the net(PVP) hydrogel (Figure 6) shows no substantial mass loss below 100 °C, indicating a low content of water and other volatile compounds. A 10% mass loss was observed at approximately 402 °C, and the maximum decomposition rate occurred at approximately 438 °C. Similarly, the net(PVP)-g-MA hydrogel exhibited no appreciable mass loss below 100 °C, indicating a low water content. However, a 10% mass loss was observed at approximately 256 °C, and two decomposition stages were identified, with maximum decomposition rates at approximately 247 and 440 °C. The first stage may be attributed primarily to the thermal degradation of the grafted MA-containing segments, whereas the second is associated with the decomposition of the PVP network. The presence of two degradation stages further supports the incorporation of MA into the hydrogel. Intermolecular hydrogen bonding between the carboxylic acid groups of MA and the carbonyl groups of PVP may also influence the thermal behavior of the modified network [25,74,80].

### 2.7. Swelling and Critical pH

**Figure 7:**
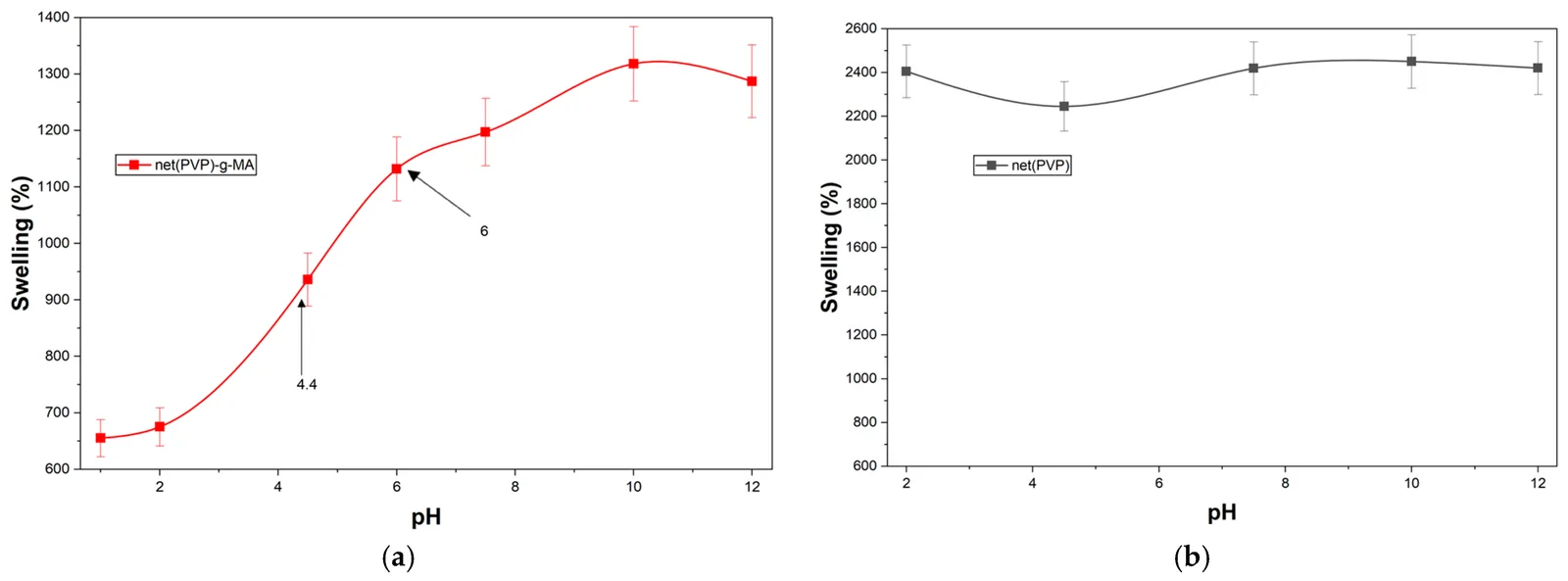
Plot of swelling vs. pH (**a**) net(PVP)-g-MA. (**b**) net(PVP).

Figure 7a shows the swelling behavior of the PVP-g-MA hydrogel as a function of pH. A progressive increase in the degree of swelling is observed as the pH of the medium increases, a behavior associated with the ionizable carboxylic acids of MA. Transition regions are identified around pH 4.45 and 6, associated with the progressive ionization of the carboxylic groups. Finally, at pH values above 10, the system approaches equilibrium. On the other hand, Figure 7b illustrates the swelling behavior of the net(PVP) hydrogel as a function of pH. Unlike its homolog net(PVP)-g-MA, net(PVP) exhibits relatively constant swelling values throughout the studied pH range, with values close to 2400–2250%, without any abrupt changes. This behavior can be attributed to the chemical nature of PVP, which lacks ionizable groups and is therefore less susceptible to pH-induced conformational changes. Additionally, the net(PVP)-g-MA hydrogel exhibits lower swelling values than those observed for net(PVP). This can be associated with modifications induced by the maleic acid graft, which could generate a more compact network and partially restrict the mobility of the polymer chains, reducing the water absorption capacity [81]. Furthermore, the grafting procedure involved a second exposure to ionizing radiation; therefore, the total cumulative dose could have induced additional changes in the PVP structure. It has been reported that PVP can undergo degradation processes at doses above 35 kGy, affecting the balance between crosslinking and chain breakage, which could contribute to the observed differences in swelling behavior [82].

### 2.8. Drug Loading and Release Kinetics

Quantifying active agents in potential burn dressings is essential for assessing the materials’ ability to incorporate and release these compounds under varying conditions, such as changes in pH and temperature. This information is useful for evaluating their potential therapeutic efficacy, determining whether adequate concentrations can be delivered to the injury site, and establishing relationships among material composition, loading capacity, and release behavior. The loading results showed that both net(PVP) and net(PVP)-g-MA hydrogels incorporated Bz-Cl and NA. However, their loading capacities varied with the chemical structure of each active agent and with maleic acid modification of the hydrogel.

**Figure 8:**
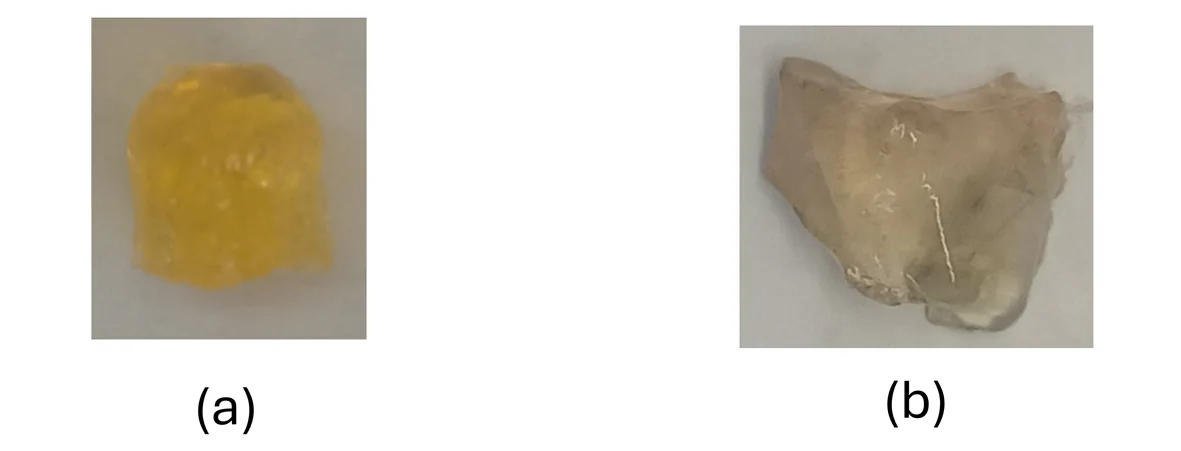

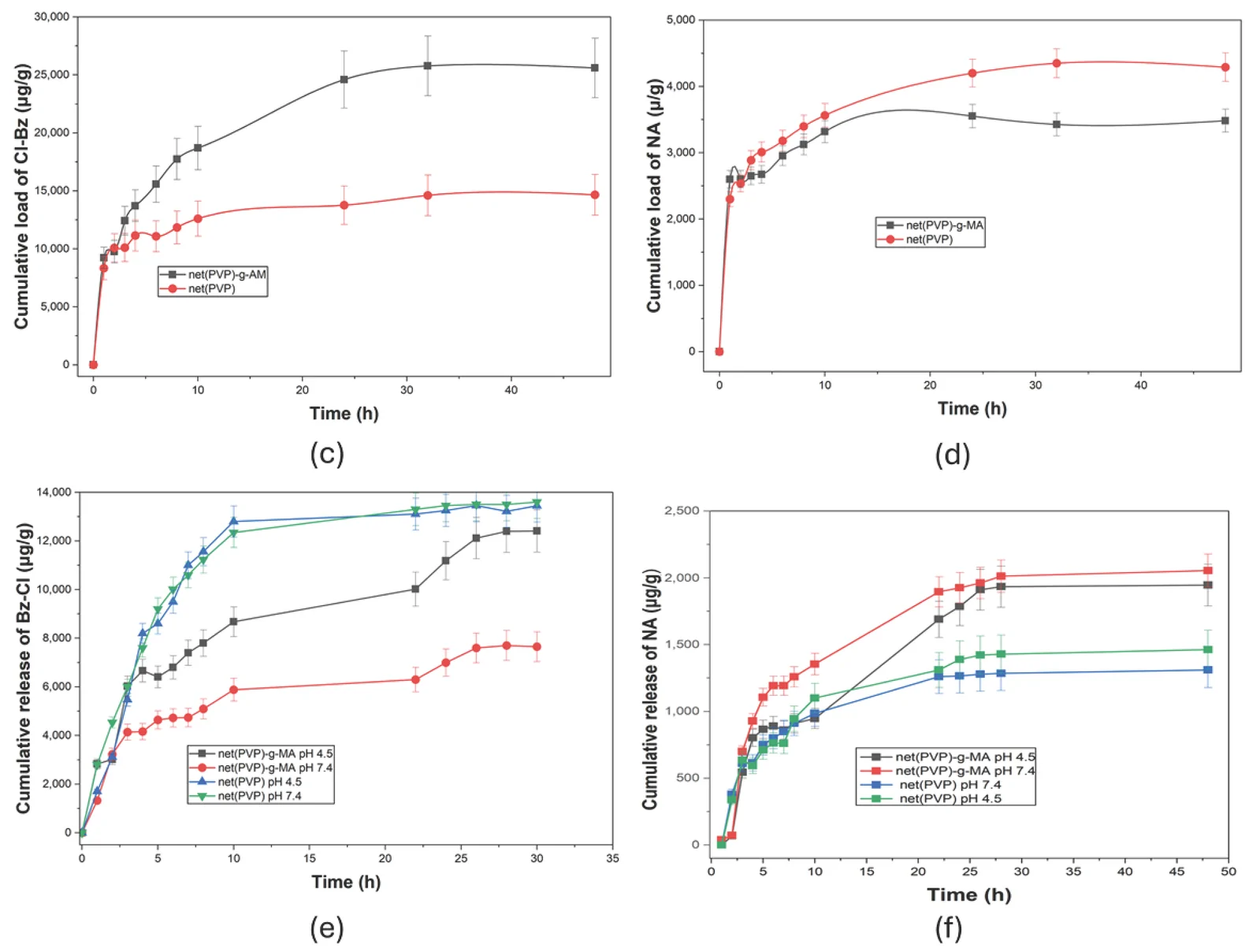
(**a**) net(PVP)-g-MA. (**b**) net(PVP)-g-AM loaded with antimicrobial silver. (**c**) Load of Bz-Cl. (**d**) Load of NA. (**e**) Release of Bz-Cl. (**f**) Release of NA.

For antimicrobial silver, quantitative determination by UV–Vis spectroscopy was not possible because the concentrations detected under the experimental conditions were below the method’s detection limit. Changes in hydrogel coloration after exposure to the silver-containing dispersion provided only qualitative evidence of silver incorporation and were therefore not used for quantitative analysis. More sensitive elemental analytical techniques, such as ICP-MS or AAS, would be required to accurately determine silver loading and release. However, loading the antimicrobial silver induced a color change, evident in the hydrogel color (Figure 8a,b).

For Bz-Cl, the net(PVP)-g-MA hydrogel achieved the highest loading capacity, approximately 25,700 µg/g (Figure 8c), compared with approximately 14,500 µg/g for net(PVP). This difference may be attributed to the cationic nature of Bz-Cl, which promotes electrostatic interactions with the carboxylate groups of maleic acid in the grafted hydrogel. Loading was performed at pH 7.4, at which a substantial fraction of the –COOH groups is ionized to –COO^−^, whereas Bz-Cl retains the positive charge of its quaternary ammonium group (R_4_N^+^). PVP may also contribute to Bz-Cl retention through ion–dipole interactions involving the carbonyl groups of its lactam rings.

In contrast, the net(PVP) hydrogel exhibited a higher NA loading capacity than the net(PVP)-g-MA hydrogel (Figure 8d). NA contains multiple hydroxyl groups capable of forming hydrogen bonds with the carbonyl groups of PVP. Although the grafted hydrogel also contains carboxylic acid groups that may interact with NA, ionization at pH 7.4 could reduce their hydrogen-bond-donating capacity and alter the hydration environment within the network. Furthermore, we hypothesized that MA grafting may produce a more compact network or steric restrictions that hinder NA diffusion into the hydrogel, thereby reducing its loading capacity.

Regarding Bz-Cl release, net(PVP) exhibited a faster and greater release than net(PVP)-g-MA, reaching approximately 13,300 µg/g after 22 h, regardless of the pH of the release medium (Figure 8e). Based on the initially loaded amount, approximately 92% of the incorporated Bz-Cl was released, indicating relatively low retention within the PVP network. This behavior suggests that the association between PVP and Bz-Cl is comparatively weak and may primarily involve ion–dipole interactions and dispersion forces. In contrast, net(PVP)-g-MA exhibited pH-dependent Bz-Cl release (Figure 8e). The lowest cumulative release occurred at pH 7.4, reaching approximately 7600 µg/g after 26 h, whereas approximately 12,400 µg/g was released at pH 4.5 after 28 h. Relative to the initially loaded amount of approximately 25,700 µg/g, these values correspond to releases of approximately 30% and 48%, respectively. The greater retention at pH 7.4 may be attributed to stronger electrostatic interactions between the ionized carboxylate groups of MA and the positively charged quaternary ammonium group of Bz-Cl. At pH 4.5, greater protonation of the carboxylic acid groups weakens these electrostatic interactions, thereby facilitating Bz-Cl release.

For NA, the greatest cumulative release was observed from the net(PVP)-g-MA hydrogel at pH 7.4, reaching approximately 2012 µg/g, followed by the same hydrogel at pH 4.5, which released approximately 1930 µg/g (Figure 8f). At pH 7.4, ionization of the carboxylic acid groups reduces their hydrogen-bond-donating capacity and increases electrostatic repulsion among the carboxylate groups, promoting network expansion and water uptake. These effects may facilitate NA diffusion and release. At pH 4.5, a greater proportion of the carboxylic acid groups remains protonated, favoring hydrogen-bonding interactions with NA and resulting in slightly lower release.

The net(PVP) hydrogels exhibited similar NA release profiles at pH 4.5 and 7.4 (Figure 8f), reaching approximately 1300 µg/g after 22 h. Their limited pH dependence is consistent with the essentially nonionic nature of PVP. The lower NA release from these hydrogels may be attributed to NA’s affinity for the PVP matrix, as its multiple hydroxyl groups can form hydrogen bonds with PVP’s carbonyl groups, favoring its retention within the polymer network and limiting its diffusion into the release medium.

Finally, none of the evaluated systems released 100% of the initially incorporated Bz-Cl or NA. This incomplete release indicates that a fraction of each active agent remained associated with or physically trapped within the polymer network due to the interactions responsible for its incorporation [83,84]. Although hydrogel swelling facilitates molecular diffusion into the surrounding medium, the release rate progressively decreases as the concentration gradient diminishes and the system approaches equilibrium. Consequently, interactions with the polymer matrix and diffusion limitations prevent complete release under the evaluated conditions.

Regarding the kinetics analyses of NA and Bz-Cl from net(PVP) and net(PVP)-g-MA hydrogels, the experimental data were fitted to several mathematical models, including the Higuchi, zero-order, first-order, Korsmeyer–Peppas, Peppas–Sahlin, and Weibull models, to identify the model that best described the release behavior of each system. The analyses were performed using the DDSolver add-in for Microsoft Excel. The goodness of fit was evaluated using three statistical parameters: the coefficient of determination (R^2^), for which values closer to 1 indicate better agreement between the experimental and predicted data; the Akaike information criterion (AIC), for which lower values indicate a better fit; and the model selection criterion (MSC), for which values greater than 2 are generally considered indicative of an acceptable fit.

Overall, the Peppas–Sahlin model provided the best fit for most systems. Analysis of the relative contributions of the Fickian and relaxation components indicated that release occurred predominantly through Fickian diffusion, with a smaller contribution from polymer-chain relaxation. The only exception was the release of Bz-Cl from net(PVP) hydrogels, for which the first-order model provided the best fit. The release kinetic parameters are presented in Table 2.

The Peppas–Sahlin model (Equation (1)) was used to estimate the relative contributions of Fickian diffusion and polymer-chain relaxation to the release process. For this purpose, the experimental data were fitted to the equation proposed by Peppas and Sahlin, which describes release from swellable polymer matrices involving anomalous transport and allows the contribution of each mechanism to the overall release process to be estimated [85].

(1) $$\frac{Qt}{Q\infty }=k_{1}t^{m}+K_{2}t^{2m}$$

where *Qt*/*Q*∞ is the fraction of drug at time *t*, *k*_1_ is the diffusion constant, *k*_2_ is the relaxation constant, and *m* is the diffusion exponent.

On the other hand, in first-order kinetics, the release suggests that the release mechanism depends on the concentration (Equation (2)). As the concentration gradient decreases with time, the amount of drug released also decreases.

(2) $$Log\;C_{1}=\log Co-kt/2.303$$

where *C*_1_ is the drug concentration at time *t*, *C*_0_ is the initial concentration, *t* is time, and *k* is the release rate.

### 2.9. Antimicrobial Studies Results

Microbiological studies are fundamental to the development of potential materials for burn treatment, as burns cause the loss of the skin’s protective barrier, promoting bacterial colonization and increasing the risk of infections that can delay healing and compromise patient recovery [86].

Evaluating against *S. aureus* and *E. coli* enables the assessment of the antimicrobial efficacy of different materials against Gram-positive and Gram-negative bacteria, which differ significantly in cell wall structure and, consequently, in susceptibility to antimicrobial agents [87]. *S. aureus* is among the most frequently isolated bacteria in wound and burn infections [88,89,90,91], whereas *E. coli* is commonly used as a representative model of Gram-negative bacteria and has also been reported to cause complications in burns [86,88,89]. Therefore, analyzing antimicrobial activity against both microorganisms provides a broader overview of the material’s potential as a burn wound dressing.

**Figure 9:**
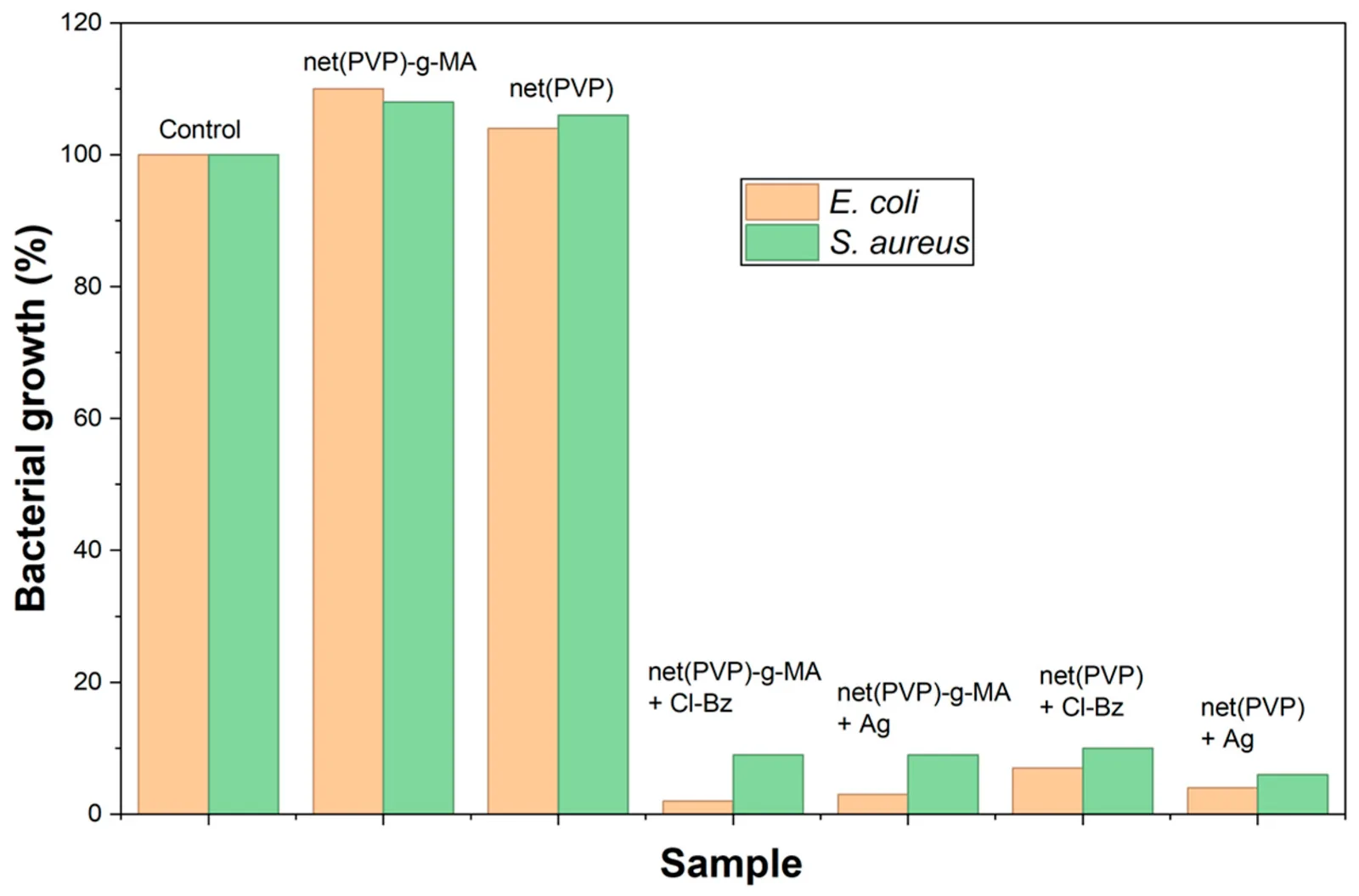
Results of microbiological tests.

The results of turbidimetric assays of the net(PVP) and net(PVP)-g-MA hydrogels loaded with Ag and Bz-Cl are shown in Figure 9. The control net(PVP) and net(PVP)-g-MA hydrogels (without Ag or Bz-Cl loading) showed no inhibition; on the contrary, a slight increase in growth (4–10%) was observed compared to the control medium. However, upon the addition of Ag or Bz-Cl to the hydrogels, growth was substantially reduced by more than 90% for both strains. To date, the mechanism of action of the synthesized silver (theoretically, AgNPs) is still not entirely clear [92]. Nevertheless, several mechanisms of action have been proposed, including:

(1) Silver ions have an affinity for groups present in proteins and cell membranes, where they form bonds that alter their structure and block essential functions. This disrupts ion transport and ATP synthesis. Furthermore, they can interact with DNA and RNA, hindering the division and reproduction of microbial cells [92].
(2) The increase in oxidative stress results from the generation of reactive oxygen species (ROS) and free radicals. These compounds can accumulate because silver promotes their formation and alters cellular antioxidant mechanisms, such as glutathione. Excess ROS can damage mitochondrial and cellular membranes, proteins, lipids, and DNA, causing DNA breakage and affecting vital cellular functions [92,93].

Regarding Bz-Cl, the mechanism of action relies on the interaction of the positively charged nitrogen of Bz-Cl with the phospholipid bilayer of the bacterial cell membrane. Furthermore, membrane destabilization causes cell lysis and leakage of intracellular components, leading to cell death [94,95,96].

## 3. Conclusions

PVP hydrogels were successfully synthesized by gamma radiation. Among the evaluated conditions, hydrogels prepared from 50% NVP in methanol at 40 kGy exhibited the highest gel content (approximately 95%) and favorable qualitative handling characteristics. Subsequent radiation-induced grafting of maleic acid reached a maximum of approximately 16.6% at 30 kGy. The incorporation of MA was supported by FTIR-ATR, DSC, and TGA analyses and introduced ionizable carboxylic acid groups into the PVP network, producing pH-responsive swelling behavior with transition regions at approximately pH 4.45 and 6.0.

On the other hand, the loading and release behavior depended on both the hydrogel composition and the chemical nature of the active agent. The net(PVP)-g-MA hydrogel showed a higher loading capacity for Bz-Cl than net(PVP), which was attributed to electrostatic interactions. In contrast, net(PVP) exhibited a higher NA loading capacity, whereas the grafted hydrogel provided greater NA release, particularly at pH 7.4. For most systems, the release profiles were best described by the Peppas–Sahlin model and were governed predominantly by Fickian diffusion, with a smaller contribution from polymer-chain relaxation.

Finally, hydrogels loaded with antimicrobial silver or Bz-Cl reduced the growth of *E. coli* and *S. aureus* by more than 90%, whereas the unloaded hydrogels showed no antimicrobial activity. Overall, these results demonstrate that radiation-induced MA grafting is an effective strategy for imparting pH responsiveness and modulating the loading and release behavior of PVP hydrogels. Therefore, the developed systems show potential as multifunctional materials for wound and burn dressings. Nevertheless, further studies are required to determine their mechanical properties and cytocompatibility and to confirm the morphology and particle-size distribution of the synthesized silver nanoparticles before their biomedical application.

## 4. Materials and Methods

### 4.1. Materials

N-vinylpyrrolidone (>99%) was purchased from Sigma-Aldrich Co. (St. Louis, MO, USA) and purified by vacuum distillation at around 110 °C. AgNO_3_ (99%) was obtained from DEQ (Nuevo León, Mexico) and was used as received. Methanol was acquired from Baker (Mexico City, Mexico). Maleic acid (>99%), polyvinylpyrrolidone (360 kDa), benzalkonium chloride, and naringin (>95%) were purchased from Sigma-Aldrich Co. (St. Louis, MO, USA).

### 4.2. Synthesis of net(PVP) Hydrogels

Hydrogels were synthesized from PVP (360 kDa) and N-vinyl-2-pyrrolidone (NVP) monomer. For this purpose, NVP solutions at different concentrations (% *w*/*w*) were prepared in methanol and water. Known amounts of these solutions were then transferred into glass ampoules, purged with Ar for 15 min, and sealed to maintain an inert atmosphere. The ampoules were subsequently irradiated at doses ranging from 10 to 60 kGy. After irradiation, the hydrogels were removed from the ampoules and washed in a methanol/water mixture for 24 h to remove the non-crosslinked polymer fraction. The hydrogels were initially dried in a fume hood, followed by drying in a vacuum desiccator for one week. Finally, they were dried in a vacuum oven at 45 °C until a constant weight was achieved. The gel content was calculated using Equation (3).

(3) $$Gel\;content\left(\%\right)=\frac{Wd}{Wi}\times 100$$

where Wd is the weight of dried hydrogel mass, and Wi is the monomer or PVP (360 kDa) initial mass, respectively.

### 4.3. Synthesis of net(PVP)-g-MA Hydrogels

The direct irradiation method was used to graft MA onto the net(PVP) hydrogels. The net(PVP) hydrogels were placed in glass ampoules, and a 35% (*w*/*v*) MA solution in methanol was added. The ampoules were then purged with Ar and sealed to maintain an inert atmosphere. Irradiation doses ranging from 10 to 60 kGy were used. After irradiation, the hydrogels were removed from the ampoules and washed in a methanol/water mixture for 24 h. They were subsequently subjected to the drying procedure described in Section 4.2. The grafting percentage was calculated using Equation (4).

(4) $$Graft\left(\%\right)=\frac{Wf-Wo}{Wo}\times 100$$

where Wf and Wo are the final weight of the grafted hydrogel and the initial weight of the non-grafted hydrogel, respectively.

### 4.4. Synthesis of Antimicrobial Silver

Antimicrobial silver was synthesized according to previously reported methods [67,68]. Briefly, 20 mL of a 0.5% (*w*/*v*) PVP solution was prepared. Subsequently, 20 mL of a 10 mM AgNO_3_ solution was added to the PVP solution under continuous stirring. The resulting mixture was transferred into test tubes and irradiated at a dose of 20 kGy. After irradiation, a UV–Vis spectrophotometric scan was performed over the wavelength range of 300–700 nm to identify the characteristic surface plasmon resonance band.

### 4.5. Psychochemical Characterization

#### 4.5.1. FTIR-ATR Analysis

Before analysis, all samples were dried at 60 °C for 72 h to eliminate residual moisture. FTIR-ATR analysis was performed using a Perkin-Elmer Spectrum 100 Spectrophotometer with a diamond tip from Perkin Elmer Cetus Instruments in Norwalk, CT, USA, performing 16 scans for each sample with the ATR module.

#### 4.5.2. Thermal Analysis

Thermogravimetric Analysis (TGA) and Differential Scanning Calorimetry (DSC) were conducted under a nitrogen atmosphere. TGA data were collected using a TA Instruments TGA Q50 (TA Instruments, New Castle, DE, USA) with a heating rate of 10 °C/min from 25 °C to 800 °C.

DSC analysis was carried out using a TA Instruments 2010 calorimeter (TA Instruments, New Castle, DE, USA). Thermal transitions were determined at a heating rate of 10 °C/min from 25 to 400 °C.

#### 4.5.3. Swelling and Determination of Critical pH

Initially, two stock solutions were prepared: (I) an acidic solution containing 0.2 M boric acid and 0.05 M citric acid, and (II) a basic solution containing 0.1 M sodium phosphate. Buffer solutions at different pH values were then prepared by mixing these stock solutions in varying ratios.

The hydrogel samples were dried at 60 °C for 24 h, and their initial weights were recorded. Each sample was then immersed in either distilled water or buffer solutions with pH values ranging from 2 to 12. At predetermined time intervals, the samples were removed from the medium, excess surface liquid was carefully blotted, and the samples were weighed. This procedure was repeated until a constant weight was reached. The swelling percentage was calculated using Equation (5), and the critical pH was determined by fitting the experimental data to the Boltzmann equation in Origin 2024. All measurements were performed in triplicate.

(5) $$Swelling\left(\%\right)=\frac{Wt-Wo}{Wo}\times 100$$

where Wt and Wo are the weight at time t and the initial weight (dry hydrogel), respectively.

### 4.6. Drug Loading and Release Studies

For the loading studies, known amounts (100–300 mg) of net(PVP) or net(PVP)-g-MA hydrogels were placed in flasks containing 30 mL of an aqueous solution of NA (30 µg/mL), benzalkonium chloride (Cl-Bz; 1 mg/mL), or antimicrobial silver (50% *v*/*v*), as appropriate. The hydrogel mass was chosen to prevent complete absorption of the loading medium. The flasks were maintained in a water bath at room temperature with continuous stirring at 60 rpm throughout the loading process. At predetermined time intervals, 3 mL aliquots were collected, and absorbance was measured using a SPECORD^®^ 200 Plus UV–Vis spectrophotometer (Analytik Jena AG, Jena, Germany) at 277 nm for NA and 257 nm for Cl-Bz. Loading was considered complete when absorbance remained constant. Quantification was performed in triplicate for each sample using Equation (6). After loading, the hydrogels were dried in a vacuum desiccator for 24 h and then used in the release studies.

(6) $$Active\;loaded=\left(\frac{Co-Cf}{W}\right)\times V$$

Cf and Co represent the final and initial concentrations of the active agent in the loading medium, respectively; *V* is the volume of the medium, and W is the dry mass of the hydrogel.

Release studies of NA- or Bz-Cl-loaded net(PVP) and net(PVP)-g-MA hydrogels were performed in triplicate at pH 4.5 and 7.4. The hydrogels were placed in glass bottles containing 30 mL of the corresponding buffer solution and incubated in a water bath at 37 °C under continuous agitation at 60 rpm. To monitor the progressive release of the active agents, 3 mL aliquots were collected at predetermined time intervals, filtered through a stainless-steel mesh with an approximate pore size of 33 µm, and transferred into 3.5 mL quartz cuvettes. After each measurement, the aliquots were returned to the release medium. NA and Bz-Cl concentrations were quantified using Equations (7) and (8), respectively, which were obtained from the corresponding calibration curves.

(7) $$Drug\;released=0.00366x+0.10832\;R^{2}=0.99113$$

(8) $$Drug\;released=0.000982934x+0.00388\;R^{2}=0.99904$$

### 4.7. Antimicrobial Studies

Mueller–Hinton culture media from BD Bioxon™ or Difco™ (Cientifica SENNA, Mexico City, Mexico) were prepared according to the manufacturers’ instructions. The required amount of each medium was weighed, and the pH was adjusted with 1.0 M HCl or 1.0 M NaOH. The media were autoclaved at 121 °C and 15 psi for 15 min, then cooled and dispensed into 5 mL tubes. Before use, sterility was verified by incubating the media at 35 °C for 24 h. *Staphylococcus aureus* (ATCC 25923) and *Escherichia coli* (ATCC 25922), preserved at −70 °C, were activated in brain heart infusion broth (BD Bioxon™) and Luria broth (BD Bioxon™), respectively, for 24 h. Subsequently, 100 µL of each culture was transferred to fresh medium and incubated for 7 h. The microbial density was adjusted to the 0.5 McFarland (MF) standard. Six consecutive tenfold dilutions of each adjusted suspension were prepared in isotonic saline solution. Aliquots of 100 µL from the 10^−4^, 10^−5^, and 10^−6^ dilutions were plated in duplicate on Mueller–Hinton agar in Petri dishes. The inoculum was spread over the agar surface, and the plates were incubated at 35 °C for 24 h. Colonies were counted to determine the bacterial concentration, yielding 1.50 × 10^8^ CFU/mL for *E. coli* and 1.8 × 10^8^ CFU/mL for *S. aureus*.

From the suspensions adjusted to the 0.5 McFarland standard, 100 µL was added to tubes containing Mueller–Hinton broth and the corresponding hydrogel samples. The tubes were incubated at 35 °C for 24 h, after which the optical density was measured at 600 nm using a spectrophotometer. Bacterial growth was evaluated using Equation (9).

(9) $$Bacterial\;growth\;inhibition\left(\%\right)=\left(\frac{A\;control-A\;sample}{A\;control}\right)\times 100$$

where A control is the absorbance of the bacterial control and A sample is the absorbance of the hydrogel-treated sample.

## Figures and Tables

**Table 1:** Gel content of net(PVP) hydrogels as a function of polymer concentration, solvent, and irradiation dose.

PVP 360 kDa (%)	Monomer (NVP) (%)	Water (%)	Methanol (%)	Dose (kGy)	Gel Content (%)
10		90		10	66 ± 5.3
10	-	90		20	72 ± 4.2
10	-	90		30	54 ± 6.5
10	-	90		40	73 ± 8.9
15	-	85		25	78 ± 3.8
15	-	85		50	81 ± 11.2
15	-	-	85	25	63 ± 4.6
15	-	-	85	50	62 ± 4.1
-	50	-	50	20	-
-	50	-	50	40	95 ± 3.3
-	50	-	50	60	83 ± 2.9

± represents standard deviation. Values are the average of 3 runs.

**Table 2:** Best-fit kinetic models for NA and Bz-Cl release from net(PVP) and net(PVP)-g-AM hydrogels.

Hydrogel	Active	pH	Model	Best Fit Parameters	R^2^	AIC	MSC
net(PVP)-g-MA	NA	7.4	Peppas–Sahlin 1 with Tlag	K_1_ 21.03 K_2_ −1.87 m 0.44 Tlag (h) 1.99	0.9946	53.135	4.656
net(PVP)-g-MA	NA	4.5	Peppas–Sahlin 2 with Tlag	K_1_ 12.22 K_2_ −0.65 m 0.57 Tlag (h) 1.86	0.9730	80.868	2.857
net(PVP)-g-MA	Bz-Cl	7.4	Peppas–Sahlin 1 with Tlag	K_1_ 9.45 K_2_ 3.04 m 0.19 Tlag (h) 0.97	0.9688	47.652	3.372
net(PVP)-g-MA	Bz-Cl	4.5	Peppas–Sahlin 1 with Tlag	K_1_ 15.72 K_2_ −0.47 m 0.36 Tlag (h) 1.99	0.9769	71.101	2.887
net(PVP)	NA	7.4	Peppas–Sahlin 2	K_1_ 12.35 K_2_ −1.02 Tlag (h) 0.68	0.9967	29.253	4.759
net(PVP)	NA	4.5	Peppas–Sahlin 2	K_1_ 12.40 K_2_ −0.93 Tlag (h) 1.280	0.9853	54.186	3.411
net(PVP)	Bz-Cl	7.4	First order with Fmax	K_1_ 0.215 Fmax (%) 92.7	0.9978	53.438	5.458
net(PVP)	Bz-Cl	4.5	First order with Tlag y Fmax	K_1_ 0.221 Tlag (h) 0.282 Fmax (%) 92.3	0.9849	86.251	3.450

## Data Availability

The original contributions presented in this study are included in the article. Further inquiries can be directed to the corresponding authors.
